# Forty years of Clar's aromatic π-sextet rule

**DOI:** 10.3389/fchem.2013.00022

**Published:** 2013-10-17

**Authors:** Miquel Solà

**Affiliations:** Institut de Química Computacional i Catàlisi and Departament de Química, Universitat de GironaGirona, Spain

**Keywords:** Clar's aromatic π-sextet rule, local aromaticity, polycyclic aromatic hydrocarbons (PAHs), benzenoid compounds, graphene

## Abstract

In 1972 Erich Clar formulated his aromatic π-sextet rule that allows discussing qualitatively the aromatic character of benzenoid species. Now, 40 years later, Clar's aromatic π-sextet rule is still a source of inspiration for many chemists. This simple rule has been validated both experimentally and theoretically. In this review, we select some particular examples to highlight the achievement of Clar's aromatic π-sextet rule in many situations and we discuss two recent successful cases of its application.

In 1931, Hückel formulated his renowned 4n + 2 rule that explain the stability of benzene as compared to cyclooctatetraene or cyclobutadiene (Hückel, 1931a, b, 1932, 1937). Since this rule is strictly valid only for monocyclic conjugated systems, several attempts were made to extend this rule to polycyclic systems. Among them, the probably most successful was Clar's π-sextet rule formulated in 1972 in the book “*The Aromatic Sextet*” (Clar, 1972). This model was inspired by the work of Armit and Robinson who were the first to use the term aromatic π-sextet (Armit and Robinson, 1925). Clar's rule states that the Kekulé resonance structure with the largest number of disjoint aromatic π-sextets, i.e., benzene-like moieties, is the most important for characterization of properties of polycyclic aromatic hydrocarbons (PAHs). Aromatic π-sextets are defined as six π-electrons localized in a single benzene-like ring separated from adjacent rings by formal CC single bonds.

Application of this rule to phenanthrene indicates that the resonance structure **2** in Scheme 1 is more important than resonance structure **1**. Therefore, outer rings in phenanthrene are expected to have a larger local aromaticity than the central ring, which in fact is observed when using different measures of local aromaticity (Schulman and Disch, 1999; Cyrański et al., 2000; Portella et al., 2005). For anthracene, the situation is different as can be seen in Scheme 2. There are three structures that have only one Clar's sextet localized in one of the three rings. The three structures are equivalent in Clar's rule and the Clar structure is better described by a superposition of these three structures. This is usually represented by the Clar structure of Scheme 2 in which the arrow indicates the existence of a migrating sextet. Because of the migrating sextet in anthracene, one can expect a similar aromaticity for the three rings and this prediction has been confirmed using several indicators of local aromaticity (Cyrański et al., 2000; Schleyer et al., 2001; Matito et al., 2005; Portella et al., 2005). Finally, Scheme 3 shows the Clar structure for triphenylene. In general, a PAH with a given number of aromatic π-sextets is kinetically more stable than its isomers with less aromatic π-sextets (Clar, 1964, 1972; Randić, 2003; Ruiz-Morales, 2004). Moreover, aromatic π-sextet rings are considered to be the most aromatic centers in the PAH. The other rings are less aromatic and are chemically more reactive (Dabestani and Ivanov, 1999).

**Scheme 1:**
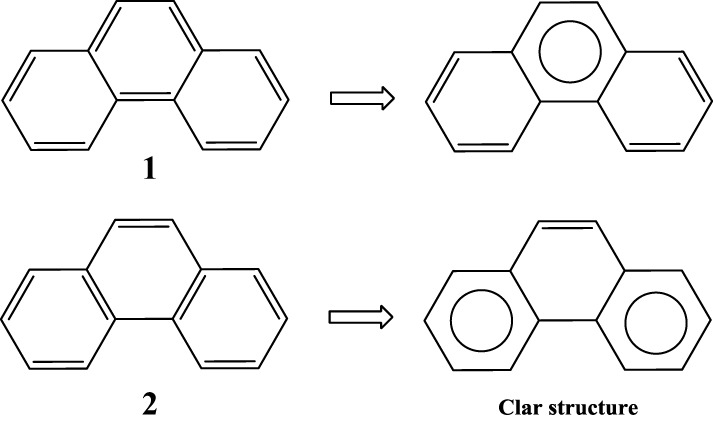
**Two (1,2) out of the five Kekulé resonance structures of phenanthrene and their corresponding Clar aromatic π-sextets indicated with a circle.** The structure with the largest number of aromatic π-sextets is the so-called Clar structure.

**Scheme 2:**
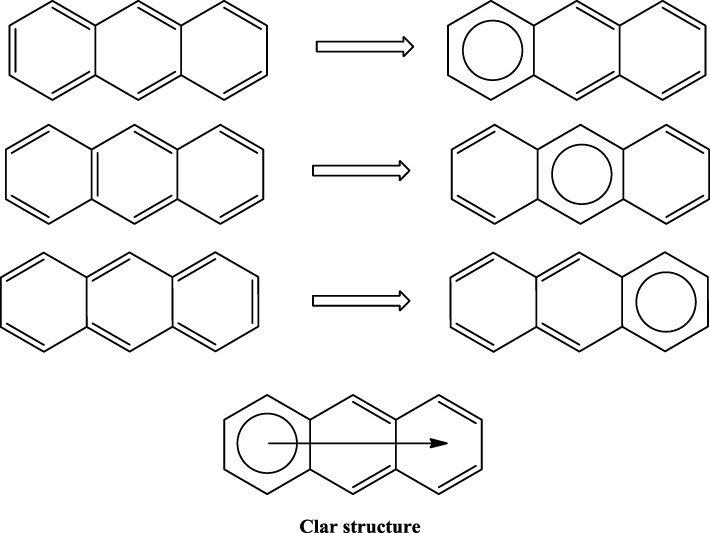
**Three out of the four Kekulé resonance structures of anthracene and their corresponding Clar aromatic π-sextets indicated with a circle.** The Clar structure has a migrating π-sextet.

**Scheme 3:**
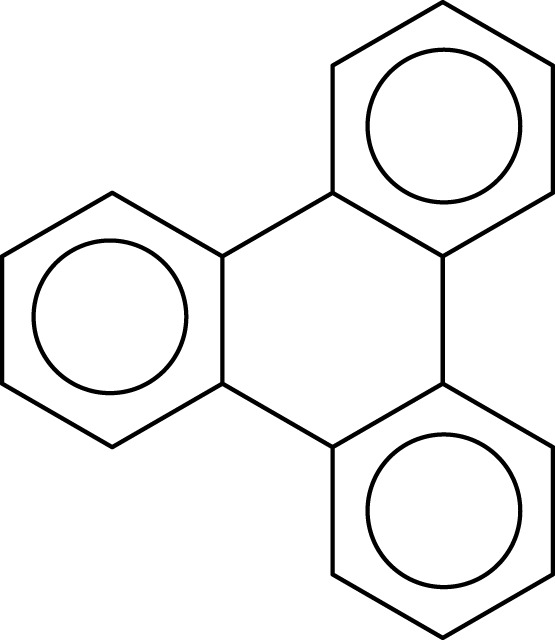
**The Clar structure in triphenylene**.

In Clar's rule one can classify the six-membered rings (6-MRs) of benzenoid species in four types of rings, namely, aromatic sextets (for instance, the phenanthrene external ring), migrating sextets (anthracene rings), empty rings (triphenylene central ring), and rings with localized double bonds (phenanthrene central ring). Similarly, one can differentiate three type of benzenoid species. First, those that have only π-sextets and “empty” rings called by Clar “fully benzenoid” (an example is triphenylene); second, those that have π-sextets and rings with a single double bond (for instance, phenanthrene); and third, those that have rings with two double bonds and for which one can write more than a single Clar structure (anthracene). Fully benzenoids were also called total resonant sextet benzenoid hydrocarbons by Dias, (Dias, 2004) all-benzenoid by Gutman et al. (Gutman and Babić, 1991) fully aromatic by Randić (2003) and Clararomatic by Balaban and coworkers (Balaban and Klein, 2009). According to Clar, fully *benzenoid* hydrocarbons having *6n* π-*electrons* (benzene, diphenyl, triphenylene…) have an extra stability (Clar, 1964, 1972; Randić, 2003). This is indeed the case of triphenylene that, among C_18_H_12_ isomers, is the one with the largest resonance energy, highest first ionization potential, largest HOMO-LUMO gap, and most chemically inert (Moran et al., 2003). Interestingly, Müllen et al. (Berresheim et al., 1999; Dötz et al., 2000) have been able to synthesize via oxidative cyclodehydrogenation of oligophenylene precursors large PAHs that are all fully benzenoid hydrocarbons. This is another indication that these fully benzenoid PAHs are particularly stable as predicted by Clar.

Multiple experimental and theoretical evidences prove that physical and chemical properties of benzenoid hydrocarbons are well-explained by Clar's aromatic π-sextet rule. From an experimental point of view, the first success of the model was to explain the reason why benzo[*qr*]naphto[2,1,8,7-*fghi*]pentacene (**3**) reacted readily with maleic anhydride, whereas its isomer tribenzo[*fg*,*ij*,*rst*]pentaphene (**4**) was unreactive (Clar and Zander, 1958). By looking at the Clar structures of **3** and **4** in Scheme 4 it becomes evident that whereas **4** is a chemically inert fully benzenoid, **3** has the possibility to act as a diene in a Diels-Alder reaction. The increased reactivity and the decrease in stability of larger acenes can also be explained by Clar's rule taking into account that the number of non-sextet rings increases along the acene series (Biermann and Schmidt, 1980; Schleyer et al., 2001; Cheng and Li, 2003; Sarova and Berberan-Santos, 2004). Moreover, the model describes the larger stability of the kinked phenacenes as compared to their linear acene isomers (Poater et al., 2007; Ciesielski et al., 2008). For instance, picene, the angular analog of pentacene, contains three aromatic sextets and is much more stable and less reactive than pentacene (with a unique migrating sextet). It is also generally recognized (Poater et al., 2007) that the 5 kcal·mol^−1^ greater thermochemical stability of phenanthrene over anthracene is related to differences in their aromaticity. Phenanthrene has two Clar π-sextets (Scheme 1) but anthracene only one (Scheme 2). Interestingly, phenanthrene and anthracene add bromine like olefins (Wiberg, 1997, 1999), the reaction rate being faster for anthracene than phenanthrene (Altschuler and Berliner, 1966). The fact that anthracene (and acenes in general) undergoes many reactions across the 9,10 position of the central ring is a consequence that by so doing two π-sextets are set up in the final adduct (compare **5** and **6** in Scheme 5) (Glidewell and Lloyd, 1984; Dabestani and Ivanov, 1999; Chien et al., 2005). In addition, there is a good correspondence with the experimental C–C bond lengths observed in X-ray structures of benzenoid compounds and the character of the C–C bonds (single, double, intermediate) predicted by the Clar structure (Wiberg, 1997; Wassmann et al., 2010). This is the case, for instance, of phenanthrene. As can be seen in Figure 1, the double bond and the single bonds of the Clar structure in Scheme 1 are the shortest and longest C–C bonds in this molecule. Another interesting confirmation comes from the UV-Vis spectra of a series of heptacatafusenes. Thus, when going from linear heptacene (one migrating π-sextet) to four kinked tetrabenzoanthracene (five π-sextets) by adding a kink (and a π-sextet) step by step one observes a reduction in the wavelength corresponding to the maximum absorption (from 840 to 326 nm) (Balaban and Klein, 2009). This demonstrates that the higher the number of π-sextets, the larger the HOMO-LUMO gap and the more stable the benzenoid is. In general, fully benzenoids have large HOMO-LUMO gaps (>2.1 eV) and are particularly stable (Chen and Liu, 2011). The ^1^H NMR spectrum of kekulene and septulene provided another validation of Clar's aromatic sextets rule (Kumar et al., 2012). Pauling's model suggested that kekulene should behave as concentric annulenes and, therefore, the inner protons should be strongly shielded. Clar's rule suggests that the ring currents are localized in the 6-MRs and, consequently, the inner protons should be deshielded. The latter prediction is the one that matches the experimental observation that inner protons resonate at δ > 7 ppm (Kumar et al., 2012). A remarkable validation of the model emerge from the scanning tunneling microscope (STM) images of PAHs showing patterns that resemble very much Clar aromatic sextet structures (Iyer et al., 1998; Ito et al., 2000; Samorí et al., 2001, 2002; Gutman et al., 2004; Watson et al., 2004). Thus, rings that have a π-sextet according to the Clar structure are more visible than those without the π-sextet. But even more impressive are the atomic force microscope (AFM) images of hexabenzocoronene revealing the expected bond order differences among two types of C–C bonds that are different according to Clar's rule as can be seen in Figure 2 (Gross et al., 2012).

**Scheme 4:**
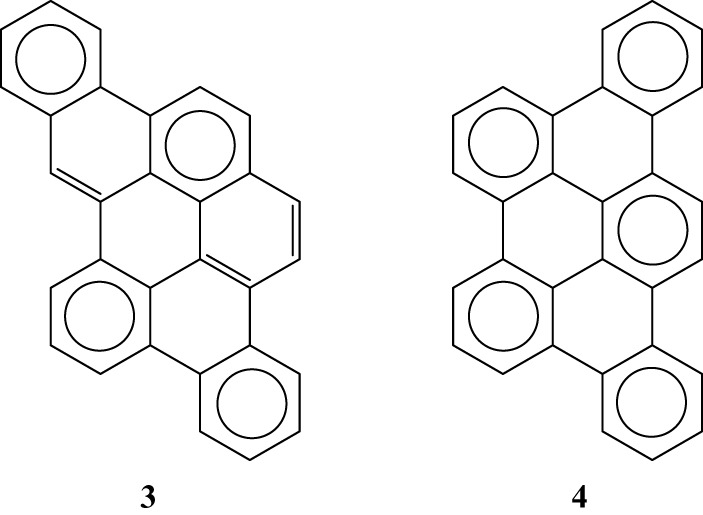
**The Clar structures of benzo[*qr*]naphto[2,1,8,7-*fghi*]pentacene (3) and tribenzo[*fg, ij, rst*]pentaphene (4)**.

**Scheme 5:**
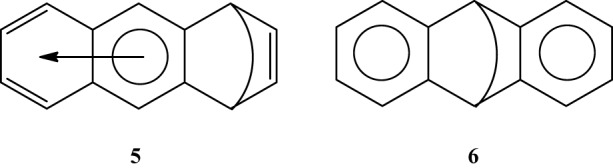
**The Clar structures of adducts obtained from a Diels-Alder addition in the external (5) or central (6) rings**.

**Figure 1:**
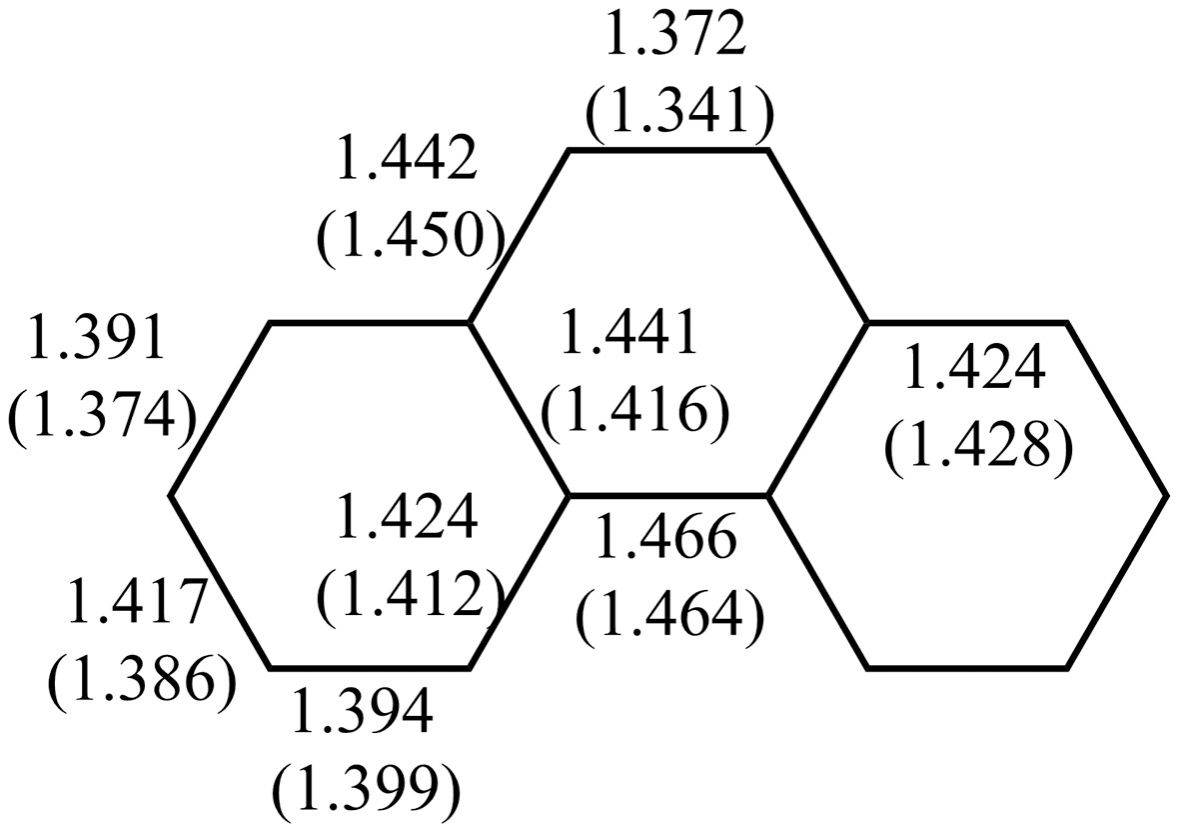
**BLYP/6-31G(d) and experimental (in parentheses)(Kay et al., 1971) bond lengths (in Å) of phenanthrene**.

**Figure 2:**
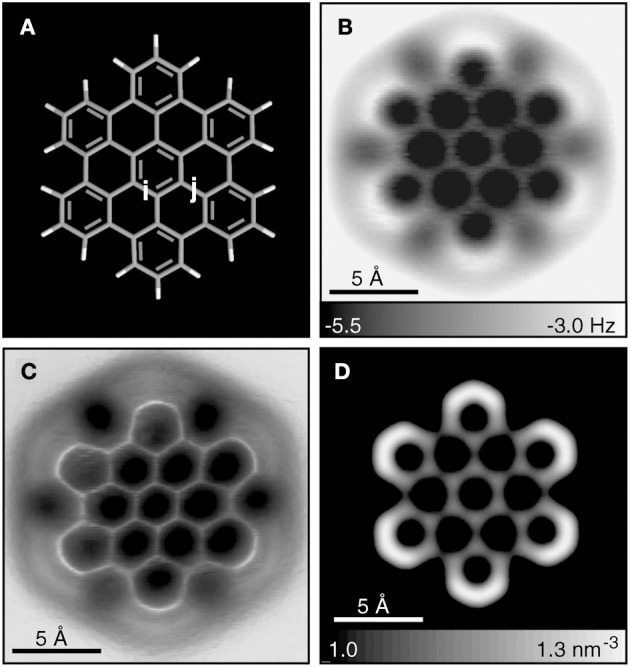
**Hexabenzocoronene model (A) and constant-height AFM measurements at *z* = 3.7 Å (B) and 3.5 Å (C). (D)** Calculated electron density at a distance of 2.5 Å. (Gross et al., 2012) Reprinted with permission from Science.

Theory and computation also provided extensive support to Clar's rule. Valence Bond calculations by Havenith et al. (2001) show that the Kékule resonance structures with the maximum number of aromatic sextets have the lowest energy and the largest contribution to the resonance energy. Nucleus independent chemical shift (NICS) (Bühl and van Wüllen, 1995; Schleyer et al., 1996; Chen et al., 2005) studies on pericondensed benzenoid PAHs point out the presence of individual aromatic rings in the positions indicated by Clar's sextet rule (Moran et al., 2003; Ruiz-Morales, 2004; Portella et al., 2005). NICS are defined as the negative value of the absolute NMR shielding computed at a ring center or at some other interesting point of the system. Rings with large negative NICS values are considered aromatic. The more negative the NICS value, the more aromatic the ring is (Schleyer et al., 1996). The conclusion from the studies on pericondensed benzenoid PAHs was reinforced through the study of ring currents in several PAHs (Steiner and Fowler, 1996; Anusooya et al., 1998; Ligabue et al., 1999; Steiner et al., 2001, 2002a, b, 2007; Aihara, 2002). Simulated STM images by Wassmann et al. (2010) clearly differentiate the Clar sextets from non-Clar hexagons in graphene ribbons. In addition, calculated HOMO-LUMO gaps for some benzenoid PAH compounds also show that, with the same number of fused aromatic rings, the PAHs with the highest number of resonant sextets present the largest HOMO-LUMO gaps (Ruiz-Morales, 2004). Finally, several local measures of aromaticity such as the *para*-delocalization index (PDI), (Poater et al., 2003) the harmonic oscillator model of aromaticity (HOMA), (Kruszewski and Krygowski, 1972; Krygowski, 1993) and NICS clearly show that these local aromaticity values calculated in benzenoid species are totally consistent with Clar's rule (Portella et al., 2005).

Clar's rule, originally developed for benzenoid compounds, has been successfully used to describe aromaticity in more complex structures as those found in carbon nanotubes, graphene nanoribbons, carbon nanocones, and carbon nanotori (Martín-Martínez et al., 2008, 2012, 2013; Balaban and Klein, 2009; Wassmann et al., 2010). Moreover, it has been proven useful to predict the coordination site of the bis(tricarbonylchromium) complex and the lithium cation to small PAHs (Güell et al.,^2005^; Jiménez-Halla et al., 2008).

As we have briefly reviewed in the previous paragraphs, the usefulness of Clar's rule has been demonstrated in many instances. For this reason, when we prepared a series of fifteen aromaticity tests that can be used to analyze the advantages and drawbacks of a group of aromaticity descriptors, (Feixas et al., 2008) we decided to include one containing five of the smallest benzenoid species that present a unique Clar structure (see Scheme 6). The position of the aromatic π-sextets in the Clar structures depicted in Scheme 6 shows the rings which are the most aromatic in benzenoid species considered according to this rule. Interestingly, all indices analyzed (PDI, FLU, MCI, I_ring_, HOMA, NICS(0), NICS(1), NICS(1)_zz_, NICS(0)_π_, NICS(0)_πzz_) indicate that rings with aromatic π-sextets are the most aromatic in the set of molecules studied (Feixas et al., 2008; Solà et al., 2010). So, this was the easiest test to pass for the indices of aromaticity checked and, in fact, it was the only one that was surpassed by all indicators of aromaticity analyzed. It is our opinion that any defined measure of aromaticity that fails to pass the test of benzenoids with a unique Clar structure should be rejected.

**Scheme 6:**
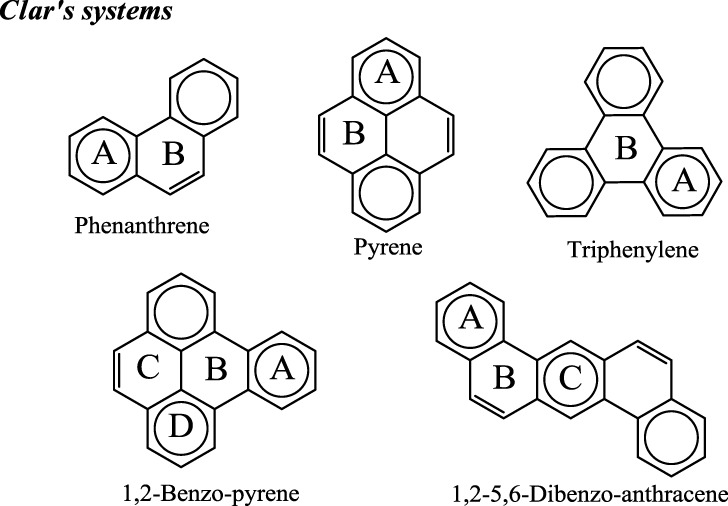
**Representation of the series of benzenoid compounds having a single Clar structure that has been included in the test series.** Clar's aromatic π-sextets are indicated with circles.

Clar's π-sextet rule is not a panacea that can solve all questions about aromaticity. It has several limitations. One of the obvious is that it provides only qualitative answers to aromaticity questions. Thus, for instance, when looking at the Clar structure of 1,2-benzo-pyrene in Scheme 6, we can safely conclude that rings A and D are more aromatic that B and C. However, we can neither establish by how much nor we can predict whether A (B) will be more aromatic than D (C) or the other way round. Another limitation is that the rule can be applied only to benzenoid species, although let us briefly mention here that an extension of Clar's rule to non benzenoid systems was proposed some years ago by Glidewell and Lloyd (1984). Moreover, there are benzenoid PAH compounds for which we cannot draw a unique Clar structure, as for instance in anthracene (Scheme 2). In this case, the method does not give an answer to which is the most aromatic ring. Moreover, some larger polybenzenoid species have many Clar structures that increase with the size of the system hindering a clear assignment of the relative aromaticity of the different 6-MRs.

Despite the numerous limitations, Clar's rule is extremely useful to explain and predict in a very simple way the structure and reactivity of many polybenzenoid compounds. However, for some reason, Clar's rule has neither received due attention nor is widely known as one can realize by looking at the syllabus of the current most renowned organic chemistry textbooks. In the following, we discuss two examples more where the Clar's π-sextet rule has been useful to analyze hydrogen bonding in benzenoid compounds and to justify changes in the ground state of acenes.

## Hydrogen bonding in o-hydroxyaryl ketones and 1,3-dihydroxyaryl-2-aldehydes

**Scheme 7:**
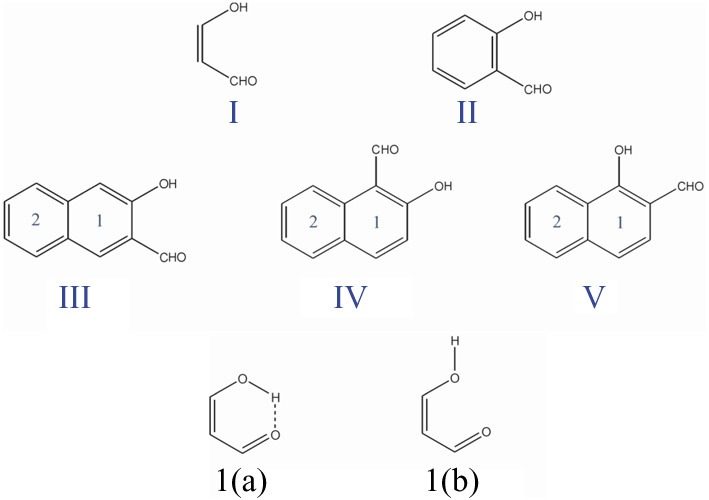
**Malonaldehyde (I), o-hydroxybenzyl aldehyde (II), and o-hydroxynaphthyl aldehyde (III, IV, and V) molecules with the corresponding labels for the different six-membered rings.** I(a) and I(b) are the closed cis and open cis conformers of malonaldehyde.

**Scheme 8:**
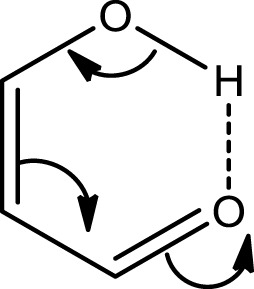
**Schematic representation of the electronic movement in the cyclic resonance assisted hydrogen bond of malonaldehyde**.

The fact that the hydrogen bond (HB) is influenced by the π-electron delocalization is well-known (Sobczyk et al., 2005). Less recognized is that with Clar's rule it is possible to predict the relative HB strength in systems having substituents in the aromatic rings connected through hydrogen bonding. In a previous work, (Palusiak et al., 2006) we analyzed a series of *o*-hydroxyaryl ketones to discuss the interrelation between the resonance-assisted hydrogen bond (RAHB) formation and the aromaticity of the adjacent aromatic rings. A subset of the systems studied is shown in Scheme 7. Usually RAHBs are found in π-conjugated rings or chain motifs, for which characteristic changes in geometrical or electronic properties are observed, i.e., elongation of formally double C–C bonds and shortening of formally single bonds together with elongation of the X-H (X = proton donor) bond and shortening of the (X)H·sY (Y = proton acceptor) bond within the H-bridge. RAHBs are HBs stronger than conventional ones whose extra stability is connected with partial delocalization of the π-electrons with the HB motif. Schematic representation of electronic effects proceeding within such cyclic RAHB is shown in Scheme 8 for malonaldehyde.

We used malonaldehyde species **I** as a reference compound since for this system there is no interaction of the RAHB with an aromatic ring. The energy difference (*E*_diff_) between optimized closed *cis* and open *cis* conformers (see **I(a)** and **I(b)** in Scheme 7) were taken as an indication of RAHB strength. For malonaldehyde the *E*_diff_ value is 13.0 kcal·mol^−1^ (see Table 1). When we move to the *o*-hydroxybenzyl ketone **II**, there is a reduction of about 2 kcal·mol^−1^ in the *E*_diff_ value. The aromatic system of π-electrons in closed *cis* **II** is relatively stable and π-electrons are less available than in **I**, which makes the communication between both substituents more difficult, reducing the effect of resonance in H-bonding in **II**. This is the reason, why the RAHB is relatively weaker in **II** as compared to **I**.

**Table 1:** B3LYP/6-311+G^**^ selected geometrical parameters usually considered as indicators of hydrogen bond strength for the closed *cis* form and energy difference between the closed *cis* and the open *cis* forms.

	***E*_diff_ [kcal·mol^-1^]**	***d*_O-H_ [Å]**	***d*_(O)H_·sO [Å]**	***d*_O_·sO [Å]**
I	12.96	0.996	1.705	2.590
II	11.07	0.984	1.765	2.638
III	10.07	0.980	1.793	2.661
IV	13.84	0.993	1.662	2.555
V	13.89	0.990	1.710	2.598

**Scheme 9:**
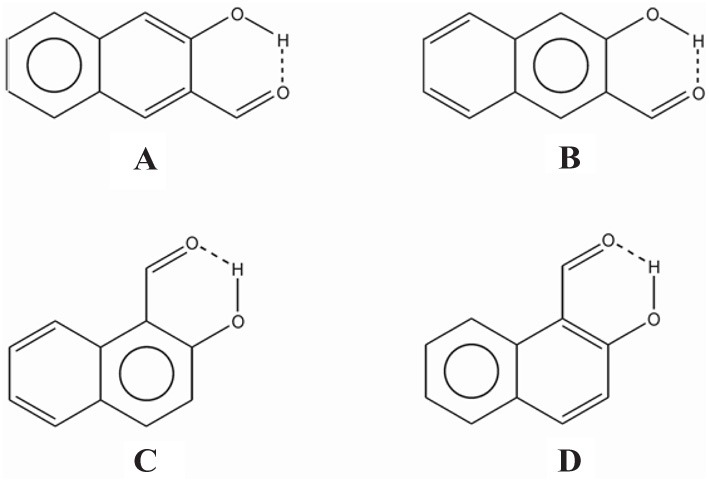
**Clar structures for species III (A and B) and IV (C and D) of Scheme 7**.

In the case of compound **III** the *E*_diff_ value is even lower than in **II**. The explanation can be found in the two possible Clar structures for **III** shown in Scheme 9. In structure **A**, the Clar π-sextet is localized within the unsubstituted ring. This implies localization of four π-electrons within substituted ring leading to a decrease of π-electrons available in the bond linking substituted carbon atoms which are those that contribute to the RAHB. In the case of Clar structure **B** this bond is significantly richer with π-electrons. This allows for supplying both the substituent effect and resonance assistance of the HB with π-electrons from the substituted ring, even if it will partially disturb the local aromaticity of this ring. So the Clar structure **B** is favored over structure **A** in the closed *cis* form of **III**, because the former helps the formation of the RAHB. Indeed, this is further confirmed by different aromaticity indices that point out a somewhat higher aromaticity of the substituted ring (despite the substitution) as compared to the unsubstituted one (HOMA: 0.756 vs. 0.738; NICS(1): −9.728 vs. −9.348; FLU: 0.012 vs. 0.012). By the way, lower FLU values indicate higher aromaticity (Matito et al., 2005). However, π-electrons available for the RAHB in **III** are less than those in reference compound **I** and, as a consequence, the RAHB in this species is also weaker than in species **I** and somewhat lower than system **II** because structure **A** also contributes to the wave function.

In the case of **IV** (and **V**), there is now a localized double CC bond linking substituted carbon atoms for structure **D**. Structure **D** is now favored as compared to **C** in the closed *cis* form since in the former the HB is assisted by the two π-electrons localized in the double CC bond linking substituted carbon atoms, while in the latter the HB is assisted by the six π-electrons which are delocalized over the whole ring and less available for assisting the HB. In this case, π-electrons available for the RAHB are similar to those in reference compound **I** and the RAHB in this species, measured by E_diff_, is stronger than in species **I** by about 1 kcal·mol^−1^. This extra stability may come from the increase in the aromatic character of ring 2 (see Scheme 7 for labels). In fact this is substantiated by different aromaticity indices that point out the lower aromaticity of the substituted ring as compared to the unsubstituted one (HOMA: 0.642 vs. 0.832; NICS(1): −8.442 vs. −10.319; FLU: 0.017 vs. 0.007).

In summary, RAHB are stronger in species for which it is possible to draw a Clar structure with a double bond accessible for π-delocalization in the RAHB (as for instance in **IV**). Less strong are the RAHB in species that can only provide a π-sextet (instead of a double bond) available for π-delocalization.

**Scheme 10:**
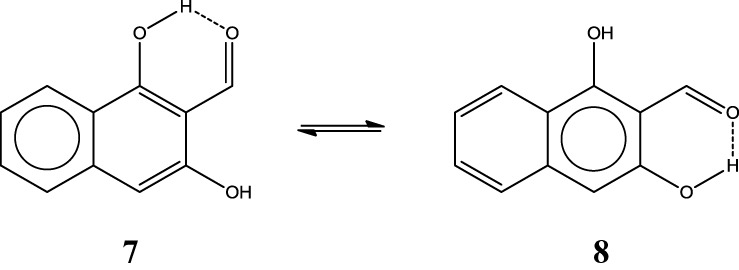
**Clar structures for two isomers (7 and 8) of o-hydroxylnaphthyl aldehyde**.

Now with this information, one can make a prediction of the HB strength in systems like the *o*-hydroxylnaphtyl aldehyde isomers **7** and **8** depicted in Scheme 10 by just drawing the Clar structures. As you may guess, **7** has a stronger HB than **8**. The difference is as large as 3.6 kcal·mol^−1^ (Palusiak et al., 2009). The reason is that in **7** we can depict a Clar structure with a double bond available for π-delocalization in the RAHB, whereas this is not possible in isomer **8**. Again the most aromatic ring in **7** is the unsubstituted one (HOMA: 0.838 vs. 0.690; FLU: 0.008 vs. 0.019; and MCI: 0.041 vs. 0.020).

## Biradical character in acenes

Acenes are PAHs consisting of linearly fused benzene rings. As the number of rings increases, the members of the acene family become increasingly reactive despite being aromatic (Yoshida and Aihara, 1999) and, as a consequence, that the higher members cannot be characterized experimentally (Schleyer et al., 2001). Although benzene and naphthalene are quite unreactive toward addition reactions, (Biermann and Schmidt, 1980) the central ring of anthracene is protonated, adds bromine, and undergoes Diels-Alder reactions readily. Tetracene and pentacene participate in even more remarkable 1,4-cycloadditions (Schleyer et al., 2001;Sarova and Berberan-Santos, 2004). The successive reduction in the band gap and reduction of the ionization potentials, as well as the increasing proton electron affinities appear to coincide with the sequential loss of benzenoid character (aromaticity) predicted by several molecular orbital treatments and Clar's qualitative sextet concept (Clar, 1964; Suresh and Gadre, 1999).

**Scheme 11:**
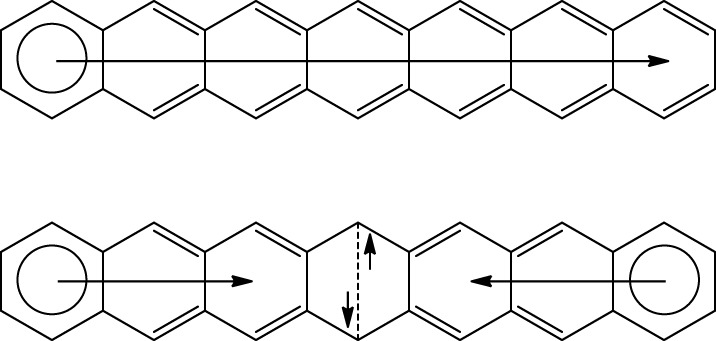
**Clar structures for the closed-shell and open-shell singlet states of heptacene**.

The electronic ground state of [n]acenes is controversial. Bendikov et al. (2004) reported that the restricted RB3LYP/6-31G(d) wave function of polyacenes longer than pentacene becomes unstable. For these species, a diradical singlet state obtained using the unrestricted broken symmetry UB3LYP/6-31G(d) method is found to be more stable than the closed-shell singlet state. Their conclusion was supported by a preliminary CASSCF investigation. Our calculations at the UB3LYP/6-31G(d) confirmed that the diradical singlet state for [n]acenes with *n* = 6–9 is more stable than the RB3LYP/6-31G(d) closed-shell singlet state by 0.1 (*n* = 6) to 6.6 kcal·mol^−1^ (n = 9) (Poater et al., 2005). For these systems, the triplet state is found slightly higher in energy than the open-shell diradical singlet state, and the energy difference between these two states decreases when n increases (Bendikov et al., 2004). The change in the electronic ground state can also be understood using Clar's π-sextet rule. As can be seen in Scheme 11, by changing from closed-shell to open-shell singlet state a π-bond is lost and this is partially or totally compensated by the formation of an extra π-sextet and some 1,4 interaction (Dewar-type resonance structure). This change is energetically unfavorable for [n]acenes with *n* < 5 or 6, but for larger *n* values is thermodynamically favorable. Large PAHs with few π-sextets have small HOMO-LUMO energy gaps that facilitate the generation of singlet or triplet open-shell ground states with biradical character (Chen and Liu, 2011). Similar arguments were employed by Einholz and Bettinger to justify the fact that dications of acenes are formed more and more easily with growing size (Einholz and Bettinger, 2013). We can add here that the concept of Clar's π-sextet was found to be germane to discuss the molecular structure and aromaticity of singlet state dicationic PAHs (Dominikowska and Palusiak, 2011).

In summary, we have illustrated the utility of Clar's rule of the aromatic sextet to predict a myriad of properties in polybenzenoid species. It is apparent from the examples discussed that, despite its limitations, Clar's rule is an intuitive, easy, and powerful tool to predict and explain many properties of sp^2^-bonded carbon materials such as benzenoid compounds or graphene nanoribbons. This is the reason why, after 40 years, Clar's aromatic sextet rule continues to catch the attention of researchers striving to understand the properties of polybenzenoid species. Surprisingly, the work by Clar is not familiar to many members of the community of organic and physical organic chemists yet. It is our hope that the current work may help to spread Clar's ideas among the chemistry community.

## Conflict of interest statement

The author declares that the research was conducted in the absence of any commercial or financial relationships that could be construed as a potential conflict of interest.
